# Green Nanotechnology-based Gold Nanomaterials for Hepatic Cancer Therapeutics: A Systematic Review

**DOI:** 10.22037/ijpr.2020.113820.14504

**Published:** 2020

**Authors:** Hamed Barabadi, Thomas J. Webster, Hossein Vahidi, Hamed Sabori, Kaveh Damavandi Kamali, Fereshteh Jazayeri Shoushtari, Mohammad Ali Mahjoub, Masoumeh Rashedi, Ebrahim Mostafavi, David Medina Cruz, Omid Hosseini, Muthupandian Saravana

**Affiliations:** a *Department of Pharmaceutical Biotechnology, School of Pharmacy, Shahid Beheshti University of Medical Sciences, Tehran, Iran. *; b *Department of Chemical Engineering, Northeastern University, Boston, MA 02115 USA. *; c *Shahid Beheshti University of Medical Sciences, Tehran, Iran. *; d *Golestan University of Medical Sciences, Food and Drug Administration, Gorgan, Iran. *; e *School of Pharmacy, Ahvaz Jundishapur University of Medical Sciences, Ahvaz, Iran. *; f *Department of Pharmaceutics, School of Pharmacy, Shahid Beheshti University of Medical Sciences, Tehran, Iran. *; g *Student Research Committee, School of Medicine, Gonabad University of Medical Sciences, Gonabad, Iran. *; h *Department of Microbiology and Immunology, Division of Biomedical Sciences, School of Medicine, College of Health Science, Mekelle University, Mekelle-1871, Ethiopia.*

**Keywords:** Nanotoxicity, Hepatic cancer, Gold nanoparticles, Biosynthesis, Anticancer activity

## Abstract

The objective of the current study was to systematically review the *in-vitro* anticancer activity of green synthesized gold nanoparticles (AuNPs) against hepatic cancer cells. The articles were identified through electronic databases, including PubMed, Scopus, Embase, Web of Science, Science Direct, ProQuest, and Cochrane. In total, 20 articles were found eligible to enter into our systematic review. Our findings showed that 65% of the articles used herbal extracts for the synthesis of AuNPs. Significantly, almost all of the articles stated the biofabrication of AuNPs below 100 nm in diameter. Impressively, most of the studies showed significant anticancer activity against HepG2 cells. Molecular studies stated the induction of apoptosis through the AuNPs-treated cells. We provided valuable information about the molecular mechanisms of AuNPs-induced cytotoxicity against HepG2 cells as well as their biocompatibility. The studies represented that AuNPs can be effective as anticancer drug nanocarrier for drug delivery systems. In addition, AuNP surface functionalization provides an opportunity to design multifunctional nanoparticles by conjugating them to diagnostic and/or therapeutic agents for theranostic purposes. Overall, our findings depicted considerable biogenic AuNPs-induced cytotoxicity, however, future studies should assess the anticancer activity of biogenic AuNPs through *in-vivo* studies, which was missing from such studies.

## Introduction

Hepatic cancer or liver cancer remains the second leading cause of cancer-related deaths and the sixth most common cancer in the world (1). The World Health Organization (WHO) anticipated that more than one million people will die from hepatic cancer in 2030, according to annual projections (2). Based on GLOBOCAN, around 782,000 new cases and 745,000 deaths of hepatic cancer occurred in 2012 in the world (3). The American Cancer Society (ACS) anticipated 42,030 new cases and 31,780 deaths, including hepatic cancer and intrahepatic bile duct cancers in 2019 in the United States. According to the ACS, the prevalence of hepatic carcinoma in American men is almost three times higher than that in American women. Moreover, it has been shown that in both males and females, the incidence of hepatic cancer is rising faster than that for all other cancers (4). 

The most common form of hepatic cancer is hepatocellular carcinoma with the most common primary malignancy of the liver comprising 70 to 85% of the total hepatic cancer burden (5). It is anticipated that 85% of hepatocellular carcinoma occurs in low and middle-income countries, especially in sub-Saharan Africa as well as Eastern Asia (6). The risk factors for hepatic cancer include obesity, fatty liver disease and diabetes, cigarette smoking, metabolic liver disease, heavy alcohol consumption, chronic hepatitis B and C viruses, and exposure to dietary toxins like aflatoxin (4, 6). Surgery is the principle therapy for patients in the early stages of the disease, while the majority of hepatic cancer cases are detected through advanced stages. Besides, sorafenib, as an FDA-approved medicine for the treatment of advanced hepatic cancer exhibits low patient survival. It was stated that in the sorafenib treatment group the overall survival was 10.7 months, while in the placebo group it was 7.9 months indicating only minimally impact patient survival of sorafenib by several months. Furthermore, chemotherapy and radiotherapy have been shown to be ineffective approaches to combat hepatic cancer (7). Thus, there is an exceeding need for a new effective therapy toward hepatic cancer. 

Nanotechnology is a new strategy to design innovative smart medicine to target hepatic cancer cells. Nanomaterials due to their specific properties can act as nanocarriers for targeted drug delivery systems (8). The diagnostic and/or therapeutic agents can be conjugated to nanomaterials for theranostic purposes (9). Among a wide range of nanomaterials, metallic nanoparticles (NPs) have attracted great interest for different applications owing to their unique optical, electrical, physical, chemical and biological properties (10-13). Metallic NPs can be synthesized through biological procedures by using natural resources ranging from plants(14-19) to microorganisms (20), micro and macroalgae (21, 22), viruses (23), and even animal tissue extracts (24). 

The biological methods are low-cost, energy-efficient, and eco-friendly (23). It was shown that in biological processes, biomolecules are responsible for reducing the metal salts to convert them to their nanoforms (25-27). These biomolecules also act as capping agents resulting in instability of these NPs (28). Among different metallic NPs, gold nanoparticles (AuNPs) have been studied significantly for a wide range of biomedical and pharmaceutical potential applications (29-32). AuNPs like their bulk counterparts are stable against oxidation under physiological conditions. Besides, the surface of AuNPs can be functionalized easily to build multifunctional NPs by a variety of ligands for therapeutic or diagnostic purposes (30).

Recently, two systematic reviews reported the efficacy of biological mediated fabricated AuNPs against colorectal and lung cancer cells through *in-vitro* investigations (33, 34). Interestingly, some of the researchers have focused on utilization of biologically synthesized AuNPs to combat hepatic cancer cells in recent years. Kalpana *et al.* synthesized AuNPs using the bacterium *Klebsiella pneumoniae* and evaluated the cytotoxicity of AuNPs in the concentrations of 0.01 to 100 µg/mL against hepatic cancer cells (HepG2) using MTT assays. After 48 h of treatment, no cell growth inhibition was observed, indicating no anticancer activity of AuNPs toward HepG2 (35). On the contrary, Shanmugasundaram *et al.* reported the synthesis of AuNPs using *Streptomyces nogalater* and evaluated the anticancer activity of AuNPs against HepG2 cells using MTT assays. The AuNPs showed significant anticancer activity against HepG2 cells after 24 h of treatment with an IC_50_ value of 43.25 µg/mL (36). 

To date, no reviews reported the anticancer activity of biogenic AuNPs against hepatic cancer. The literature is suffering from a comprehensive review to evaluate the cytotoxicity of biogenic AuNPs toward hepatic cancer cells. Due to the importance of finding new strategies for hepatic cancer therapy, we conducted a global systematic review through original published articles to evaluate the anticancer activity of biogenic AuNPs against hepatic cancer cells. In this systematic review, we discussed the different cytotoxicity of biogenic AuNPs against HepG2 cells and also highlight the challenges that should be addressed for the translation of laboratory setting studies to clinical trials. Furthermore, we discuss the proposed molecular mechanisms of the anticancer activity of biogenic AuNPs against hepatic cancer cells. 

## Experimental

This investigation is a systematic review representing the anticancer activity of green AuNPs toward hepatic cancer cells. 

*Data Source and Search Strategy*

The articles that were published up to September 25, 2019, were identified through online databases comprising of PubMed, Scopus, Embase, Web of Science, Science Direct, ProQuest, and Cochrane. The keywords included “Au”, “gold”, and “biofabrication”, “biosynthesis”, “synthesis”, “fabrication”, “microbial”, “plant*”, “biological”, “herbal”, “biomimetic”, “fungal”, “biogenic”, “green”, “bacterial”, “myco*”, “alga*”, “phyto*”, “bioreduction”, and “nano-gold”, “nanoparticle*”, “colloidal”, “nanostructure*”, “nanomaterial*”, and “antineoplastic”, “cytotoxicity”, “cancer*”, “cytotoxic”, “cell line*”, “tumor*”, “antitumor*”, “anticancer*”, “hepatic”, “liver”. 

*Inclusion Criteria*

We included articles that met the following criteria: a) published peer-reviewed articles; b) research articles published up to 25 September 2019; c) English language articles; d) original *in-vitro* studies; e) articles containing sufficient data; and f) articles evaluating the cytotoxic influence of green AuNPs against hepatic cancer cells.

*Exclusion Criteria*

We excluded the articles that met the following criteria: a) articles not in the English language; b) articles containing inadequate information; c) review articles; d) letters to the editor; e) editorials; f) congress abstracts; g) articles evaluating the cytotoxic influence of chemical and/or physical mediated fabrication of AuNPs against hepatic cancer cells; and h) reports evaluating the cytotoxic impact of green AuNPs against any other cell lines except hepatic cancer cells. 

*Eligibility Assessment*

The guidelines of Preferred Reporting Items for Systematic Reviews and Meta-analyses (PRISMA) were used for eligibility assessment of the identified articles (37). The articles were screened through first and second screening. The first screening was conducted by reviewing the articles’ titles and abstracts. In this step, most of the irrelevant articles were excluded. Then, for the remained articles, the second screening was conducted by reviewing their full texts to select the eligible articles that met all inclusion characteristics. Two individual researchers performed the eligibility assessments to avoid bias. For the case of disagreement, a third one judged. 

*Data Extraction and Tabulation*

Two individual researchers extracted the information from the selected articles using a data extraction form containing first author, year of publication, a biological source with scientific name, characterization techniques, size (nm), morphology, hepatic cancer cell line, dose, exposure time, cytotoxicity method, and significant outcome (Table 1).

## Results

*Search Results*

**Figure 1:**
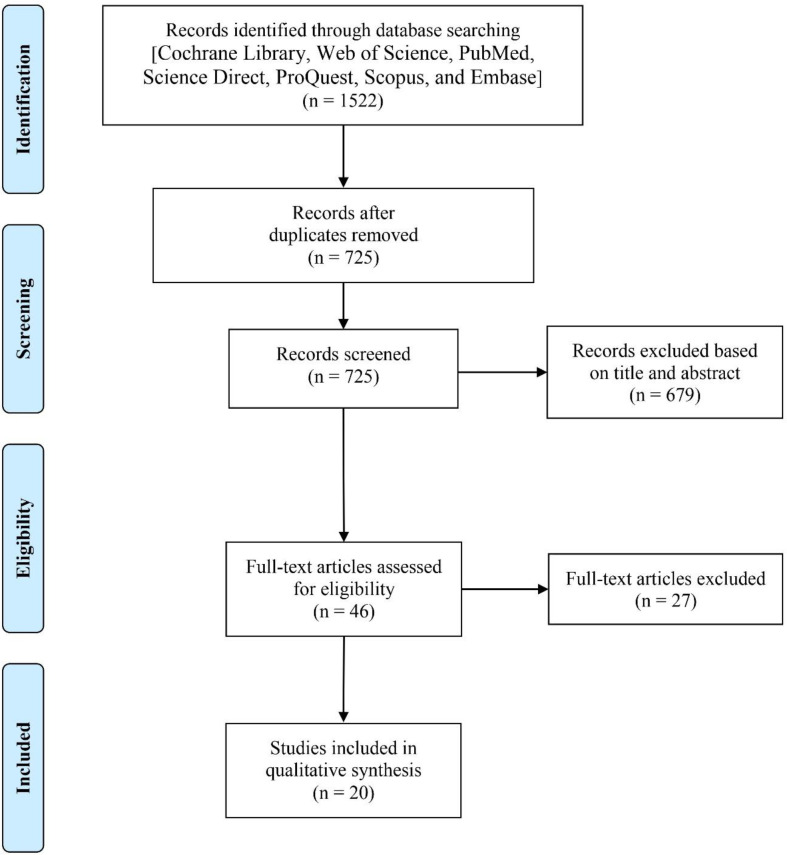
Flowchart describing the study design process

Of 1522 identified records, 797 records found a duplicate. 679 articles were excluded through the first screening and 27 articles were excluded through the second screening. Finally, 20 articles were found eligible to enter into the current study. The search process is summarized in Figure 1.

*Characteristics of Included Studies*

Our findings showed that 65% of articles applied plant extracts for the fabrication of AuNPs as reducing and capping agents (38-50). However, bacteria (35, 36), algae (51-53) and fungi (54, 55) were other natural sources that were applied for the preparation of AuNPs. The size distribution of AuNPs in almost all of the articles were found to be less than 100 nm in diameter. Spherical shaped AuNPs were fabricated in most of the articles. Cytotoxicity studies was performed by using MTT (3-(4,5-dimethylthiazol-2-yl)-2,5-diphenyltetrazolium bromide) assays in all of the articles. Moreover, the cytotoxic investigation was performed toward HepG2 in all of the articles. A vast 80% of articles (36, 38-45, 47-50, 52, 53 and 55) stated significant anticancer activity of green AuNPs against HepG2 cells. Whereas 15% (46, 51 and 54) and 5% (35) of articles reported less AuNPs-induced cytotoxicity, and no cytotoxicity against HepG2 cells, respectively.

*Strategies Used to Synthesize Nanomaterials*

The bottom-up and top-down approaches are two basic strategies for the synthesis of nanomaterials. In the top-down approach, the nanomaterials are fabricated from their bulk-state counterparts. The milling technique is an example of a top-down approach that cuts down the bulk materials to reach their fine particles (56, 57). In the bottom-up approach, the process starts at the molecular level, and the collection of these molecules finally results in the formation of nanoparticles (58). The aggregation of nanoparticles in the bottom-up approach is a challenging concern. Extensive research has been performed to prevent the aggregation of nanomaterials during the synthesis procedures by using stabilizing and/or capping agents (59-61). Chemical reduction and biosynthesis are two methods that are classified into a bottom-up approach. In chemical reduction, the existence of a reducing agent in the procedure is not adequate alone and external additive stabilizing agents like polymers is necessary to avoid aggregation (62, 63), whereas, in the biosynthetic approach, the biomolecules play a role as reducing and stabilizing agents (64). The chemical methods may use toxic chemicals that may be harmful to the environment and human, while the biosynthetic approach is eco-friendly and non-toxic for the preparation of nanoparticles (65-67). 

**Figure 2:**
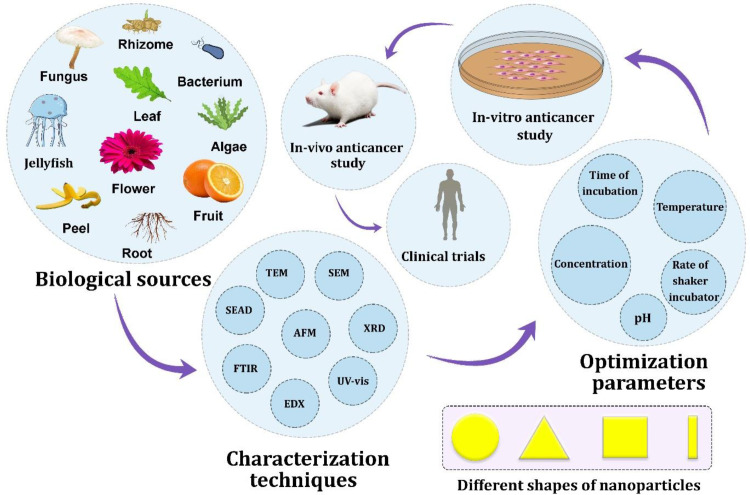
The interface of nature, nanotechnology and hepatic cancer

**Figure 3:**
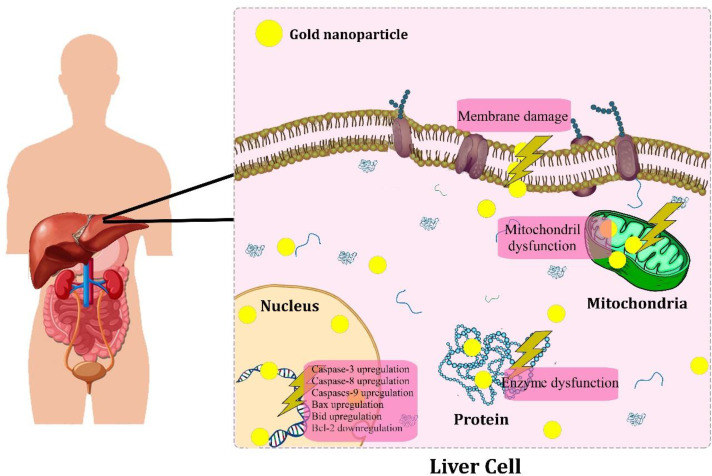
Schematic anti-cancer mechanisms of biogenic AuNPs against hepatic cancer

**Table 1:** The results of the anticancer activity of biosynthesized AuNPs against hepatic cancer cells

Ref.	Major outcome	Method	Exposure time (h)	Dose	Hepatic cancer cell line	Size (nm)/Morphology	Characterization techniques	Biological source/Scientific name	Author/Year
**(** 38 **)**	IC_50_: 70.2 ng/mL	MTT	No data	30-300 ng/mL	HepG2	Average: 20 nm/Multi-shaped, predominantly pseudo-spherical	UV-vis, HR-TEM, AFM, SAED, DLS,	Plant/*Citrus macroptera*	Majumdar *et al. *(2019)
**(** 39 **)**	IC_50_: 59.62 ± 4.37 µg/mL	MTT	24	5-100 µg/mL	HepG2	30-50 nm/Spherical and oval-shaped	UV-vis, XRD, FT-R	Plant/*Marsdenia tenacissima*	Li *et al. *(2019)
**(** 40 **)**	The IC_50_ value was 127.1 μM for nanospheres, 81.8 μM for nanostars, and 22.7 μM for nanorods.	MTT	24	31.25-500 µM	HepG2	(Average: 8.7 ± 1.7 nm/Spherical); (Average length:60.4 nm; Average width: 16.4 nm/Rod); (Average: 99 ± 47 nm/Star)	UV-vis, HR-TEM, FT-IR	Plant/Green tea	Lee *et al. *(2019)
**(** 55 **)**	IC_50_: between 10 to 12.5 µg/mL	MTT	24	2.5-15 µg/mL	HepG2	15-25 nm/Spherical	UV-vis, HR-TEM, XRD, FT-IR	Fungus/*Cordyceps militaris*	Ji *et al. *(2019)
**(** 41 **)**	100 µg/mL of plant extract plus 100 µg/mL of AuNPs led to around 60% cell growth inhibition.	MTT	24	100 µg/mL	HepG2	40-80 nm/Spherical	UV-vis, FT-IR, SEM, XRD	Plant/*Euphorbia peplus*	Ghramh *et al. *(2019)
**(** 42 **)**	IC_50_: 31 µg/mL	MTT	24	50-150 μg/mL	HepG2	Less than 20 nm/Spherical, rod and triangular	UV-vis, SEM, XRD, DLS, HR-TEM, FT-IR	Plant/*Coleus aromaticus*	Boomi *et al. *(2019)
**(** 44 **)**	IC_50_: 108.21 µg/mL	MTT	24	50-1000 µg/mL	HepG2	Average: 8.4 ± 0.084 nm/Spherical	UV-vis, XRD, FT-IR, TEM, FT-IR, SAED, EDX, FE-SEM, Zeta potential	Plant/*Backhousia citriodora*	Khandanlou *et al. *(2018)
**(** 45 **)**	IC_50_: 10.3 µg/mL	MTT	24	1.56-50 µg/mL	HepG2	37–50 nm/Quasi-spherical	UV-vis, TEM, XRD, TGA, FT-IR	Plant/*Corchorus olitorius*	Ismail* et al. *(2018)
**(** 54 **)**	33.5% of cell growth inhibition was found.	MTT	48	25 µg/mL	HepG2	10-30 nm/Spherical	UV-vis, XRD, TEM, FT-IR, Zeta sizer	Fungus/*Pleurotus ostreatus*	El Domany *et al.* (2018)
**(** 46 **)**	More than 60% of cell viability was found at 100 µg/mL.	MTT	48	10-100 µg/mL	HepG2	10-30 nm/Spherical	UV-vis, HR-TEM, FT-IR, XRD, DLS	Plant/*Coleus forskohlii*	Dhayalan *et al. *(2018)
**(** 43 **)**	IC_50_: 2.97 µg/mL	MTT	48	3.9-500 µg/mL	HepG2	Average: 47 nm/Mostly spherical	XRD, FTIR, SEM, TEM, DLS, EDX, and SAED	Plant/*Olax nana* Wall. ex Benth.	Ovais *et al.* (2018)
**(** 47 **)**	IC_50_: 20 µg/mL	MTT	72	10-100 µg/mL	HepG2	30-110 nm/flower shape	HR-TEM, FE-SEM, UV-Vis, FT-IR, XRD, XPS, TGA	Plant/*Syzygium cumini*	Borah *et* *al.* (2018)
**(** 36 **)**	IC_50_: 43.25 µg/mL	MTT	24	6.25-100 µg/mL	HepG2	Average: 14.5 nm/Spherical	XRD, FT-IR, SAED, TEM, XPS	Bacterium/*Streptomyces nogalater*	Shanmugasundaram *et al.* (2017)
**(** 51 **)**	47% cell growth inhibition was found at 100 µg/mL.	MTT	No data	1-100 µg/mL	HepG2	Average: 8-10 nm/Spherical	XRD, FTIR, SEM, TEM, EDX, SAED	Alga/*Padina tetrastromatica*	Rajeshkumar *et al.* (2017)
**(** 48 **)**	IC_50_: 100 µg/mL for CP-AuNPs; IC_50_: 150 µg/mL for CR-AuNPs	MTT	24	10-250 µg/mL	HepG2	3.5-9 nm/Mostly spherical and few triangle and hexagonal	UV-vis, XRD, FT-IR, SEM, HR-TEM, SAED	Plant/*Carica papaya* (CP) and *Catharanthus roseus* (CR)	Muthukumar *et al.* (2016)
**(** 49 **)**	IC_50_: 30 µg/mL	MTT	24	5-100 µg/mL	HepG2	25-35 nm/Predominantly spherical	HR-TEM, EDX, XRD, FT-IR	Plant/*Cassia roxburghii*	Balashanmugam *et al.* (2016)
**(** 52 **)**	IC_50_: 7.14 ± 1.45 µg/mL	MTT	72	0-100 µg/mL	HepG2	2-8 nm/Spherical	UV-vis, TEM, SEM, EDX	Alga/*Sargassum glaucescens*	Ajdari *et* *al.* (2016)
**(** 53 **)**	IC_50_: 51.9 nM	MTT	48	5-80 nM	HepG2	9-21 nm/Nearly spherical and few triangular and hexagonal	UV-Vis, DLS, HR-TEM, EDX, Zeta potential	Alga/*Padina gymnospora*	Singh *et al. *(2015)
**(** 35 **)**	No cytotoxicity	MTT	48	0.01-100 µg/mL	HepG2	16-50 nm/Spherical	UV-vis, TEM, XRD, FT-IR	Bacterium/*Klebsiella pneumoniae*	Kalpana *et al. *(2014)
**(** 50 **)**	IC_50_: 6 µg/mL after 24 h of treatment; 20% cell viability was found at 2 µg/mL after 48 h of treatment.	MTT	24, 48	2-10 µg/mL	HepG2	9-41 nm/Spherical	UV-vis, TEM, XRD, SAED	Plant/*Cajanus cajan*	Ashokkumar *et al. *(2014)

*Emerging Nano-biomaterials: A Biosynthetic Approach*

Emerging nano-biomaterials and in particular, biogenic metallic nanoparticles, have been one of the most challenging and fastest-growing sectors of nanotechnology throughout the world over the last decade (43, 56, 58, 65 and 67). The biosynthesis of metallic nanoparticles has been accomplished using a wide range of biological resources ranging from plants and algae to microorganisms such as bacteria, fungi, actinomycetes, and yeast (10). Each plant and/or algae, as well as species from each type of microorganisms, can synthesize the same composition of metallic nanoparticles with a different size distribution and morphology such as a sphere, triangular, cubic, rod, *etc.* (33, 34). Microorganisms may synthesize metallic nanoparticles intracellularly or extracellularly. In the intracellular pathway, the metal ions are transferred into the microorganism to convert to their nanoforms in the presence of biomolecules and enzymes.

In contrast, in the extracellular pathway, the extracellular secreted enzymes and biomolecules in the medium are responsible for converting the metal ions to their nanoforms (68). Besides, the plant-mediated synthesis of metallic nanoparticles consists of the use of a plant extract as a reducing and capping agent for converting metal ions to their nanoforms (65). The phytosynthesis of metallic nanoparticles seems to be more attractive compared to microbial synthesis (34, 56 and 58). 

The findings from this systematic review also confirmed phytosynthesis as a general approach for the synthesis of AuNPs. The metallic nanoparticles having a size distribution of less than 100 nm exhibited unique physical, chemical, optical, and biological properties that are not exhibited by their bulk-state counterparts (10). In a biosynthetic approach for the fabrication of metallic nanoparticles, several parameters influence the unique properties of nanoparticles such as type of biological sources, pH, temperature, reaction medium, surface charge, *etc.* (33, 34). Optimizing these parameters results in the control of size, shape and monodispersity of these nanoparticles. Then, in each optimized condition, the behavior of nanoparticles can be determined. This is the reason that elucidates the high number of publications during the past decade that applied different biological procedures for the fabrication of metallic nanoparticles. 

*Bioactivity of Gold Nano-biomaterials Compared to Chemical-mediated Synthesized Gold Nanomaterials*

Gold nano-biomaterials represented a wide range of bioactivites and have the potential to be utilized for various biomedical and pharmaceutical applications (69). In biosynthetic approach for NPs fabrication, biological molecules are responsible for bioreduction and stabilization of metallic NPs, and surround these NPs as capping agent. These biomolecules may improve the bioactivity of green synthesized metallic NPs (70). Interestingly, a study reported that hesperetin capped AuNPs improved the treatment of hepatocellular carcinoma in male Wistar albino rats and reduced the dose of chemotherapy drug. More interestingly, hesperetin capped AuNPs represented significant anti-inflammatory and anti-proliferative activity during liver carcinogenesis (71). The studies reported different bioactivity of biologically and chemically synthesized AuNPs. For instance, in a study, curcumin capped AuNPs showed higher antioxidant activity than citrate capped AuNPs (72). In another study, plant-mediated synthesized AuNPs showed significantly more cytotoxicity than chemically synthesized AuNPs against different cancer cell lines (HeLa, MCF-7, A549 and H1299). The IC_50_ value of biosynthesized AuNPs was found approximately 200 µg/mL for all cancer cells, whereas no cytotoxicity was observed at 400 µg/mL (the maximum dose studied) against healthy human embryonic kidney cells. For the case of chemichally fabricated AuNPs, approximately 20 to 25% cytotoxicity was found against all cancer cells at 400 µg/mL (the maximum dose studied) as well as a dose dependent cytotoxicity against healthy human embryonic kidney cells (73). 

*Anti-cancer Gold Nano-biomaterials to Combat Hepatic Cancer Cells*

Our findings showed the significant anticancer potential of biogenic AuNPs against hepatic cancer cells through *in-vitro* models. Figure 2 shows the interface of nature, nanotechnology and hepatic cancer. According to Table 1, a variation was observed between the cytotoxicity responses. This refers to the different natural sources that were used for the biosynthesis of AuNPs, the size distribution, the morphology, the situation of the study and even the skills of the researchers to perform the cytotoxicity assay. It has been shown that the unique physicochemical and biological properties of the nanoparticles differ by a change in their characteristics, such as shape and size. Hence, the difference in the half-maximal inhibitory concentration (IC_50_) is due to the direct influence of variation in nanoparticle characteristics among all studies. The lower the IC_50_ values, the more the cytotoxicity of the sample. According to the study of Lee *et al.*, the anticancer activity of biogenic AuNPs against HepG2 cells is shape-dependent. After 24 h of treatment of HepG2 cells with biogenic AuNPs using MTT assays, the IC_50_ value was found at 127.1 μM for nanospheres, 81.8 μM for nanostars, and 22.7 μM for nanorods. This means that nanorods (average length size: 60.4 nm; average width size: 16.4 nm) were more cytotoxic than nanostars (average size: 99 ± 47 nm) and nanospheres (average size: 8.7 ± 1.7 nm) against HepG2 cells (40).

Moreover, the nanostars were more cytotoxic than nanospheres against HepG2 cells. Furthermore, in another study, the cytotoxicity of AuNPs synthesized from two different plants was evaluated toward HepG2 cells using MTT assays. The AuNPs were synthesized by two plants, including *Carica papaya* (*CP*) and *Catharanthus roseus* (*CR*), separately. The anticancer activity of the *CP*- and *CR*-mediated fabricated AuNPs against HepG2 cells after 24 h of treatment showed significant AuNPs-induced cytotoxicity with an IC_50_ value of 100 µg/mL for *CP*-mediated synthesized AuNPs and an IC_50_ value of 150 µg/mL for the *CR*-mediated synthesized AuNPs. The comparison of IC_50_ values in this study revealed that the AuNPs synthesized from* Carica papaya* as more cytotoxic than that synthesized from *Catharanthus roseus* (48). 

Further, a time-dependent cytotoxicity of biogenic AuNPs against HepG2 cells was reported by Ashokkumar *et al.* They fabricated AuNPs using the plant extract of *Cajanus cajan *with a size distribution ranging from 9 to 41 nm and spherical morphology. They evaluated the phytofabricated AuNPs-induced cytotoxicity against HepG2 cells in the range of 2 to 10 µg/mL using an MTT assay after 24 and 48 h of treatment. After 24 h of treatment, the IC_50_ value was found at 6 µg/mL, and after 48 h of treatment, at 2 µg/mL (minimum concentration that was studied), 80% of growth inhibition was observed which indicated a time-dependent cytotoxicity of biogenic AuNPs against HepG2 cells (50). 

Studies have also shown that AuNPs could act as a nanocarrier for drug delivery systems. In a study, pectin was applied for the fabrication of AuNPs as a reducing and capping agent. The synthesized pectin-capped AuNPs were found to be spherical with an average diameter size of 14 nm. The anticancer drug doxorubicin was loaded onto the synthesized AuNPs. Drug loading was found at 78% with high stability under different electrolytic conditions and varying pH. Then, the anticancer activity of free doxorubicin was compared to doxorubicin-loaded AuNPs and also free AuNPs against HepG2 cells. The results showed that the pectin-capped AuNPs without doxorubicin had no cytotoxic influence toward HepG2 cells. Besides, the doxorubicin-loaded AuNPs were found to be more cytotoxic than free doxorubicin toward HepG2 cells (74). This can be evidence indicating the potential of AuNPs as a promising nanocarrier for anticancer drug delivery to overcome hepatic cancer.

*Mechanistic Approach for the Anti-cancer Activity of Gold Nano-biomaterials Toward* *Hepatic Cancer Cells*

Studies proposed the possible molecular mechanisms of biogenic AuNPs-induced cytotoxicity in hepatic cancer cells. Figure 3 represents a schematic anti-cancer mechanisms of biogenic AuNPs against hepatic cancer. In a study, the anticancer activity of phytosynthesized AuNPs was reported against HepG2 cells with an IC_50_ value of 59.62 ± 4.37 µg/mL after 24 h of treatment through MTT assays. The western blotting technique was used to assess the cytotoxic influence of AuNPs on the apoptotic signaling proteins in HepG2 cells. The results showed the upregulation of the expression of pro-apoptotic Bax, caspase-9 and caspase-3 proteins. In contrast, the expression of anti-apoptotic Bcl-2 and Bcl-XL proteins were downregulated, indicating the activation of the apoptosis pathways in the AuNPs-treated cells. In contrast, the untreated cells showed increased expression of anti-apoptotic proteins (Bcl-XL and Bcl-2). Moreover, intracellular reactive oxygen species (ROS) in the AuNPs treated Hepg2 cells was determined using the dichlorofluorescein diacetate staining method. The results showed high fluorescent intensity in the treated cells compared to control, indicating overgeneration of intracellular ROS through AuNPs-treated cells (39). 

In a similar study, the significant anticancer activity of mushroom-mediated fabricated AuNPs was reported against HepG2 cells. The results confirmed elevated intracellular overgeneration of ROS. Besides, the real-time PCR analysis showed that in AuNPs-treated cells, the apoptotic genes, including caspase-9, caspase-3, Bax and Bid expression were upregulated, whereas the expression of anti-apoptotic Bcl-2 was downregulated. Moreover, the induction of apoptosis was evaluated by quantifying the enzyme activity of caspases-3, -8, and -9 through the AuNPs-treated cells by using a colorimetric assay kit method. The findings showed that the enzyme activity of the apoptotic caspases-3, -8 and -9 dramatically increased in the treated cells compared to untreated cells. Besides, the rhodamine-123 staining method was performed to evaluate the integrity of the mitochondrial membrane potential (MMP) in treated HepG-2 cells. The results showed that the integrity of MMP was disrupted in the AuNPs-treated cells (55). 

Additionally, in a study, the DNA fragmentation assay (as a characteristic feature of apoptosis) was performed to evaluate the DNA damage in the biogenic AuNPs-treated HepG2 cells. The results showed fragmented DNA in the treated cells compared to the control. Besides, flow cytometry analysis revealed the presence of apoptotic cells in AuNPs-treated cells (36). In a similar study, the fragmented DNA was confirmed in biogenic AuNPs-treated cells by using a DNA fragmentation assay. In apoptosis, the chromosomal DNA cleaves into oligonucleosomal fragments (49). Furthermore, in another study, the spherical AuNPs were phytosynthesized in the size distribution ranging from 9 to 41 nm, and consequently, the anticancer activity of AuNPs was evaluated against HepG2 cells. The AuNPs showed significant anticancer activity. The oxidative DNA damage was assessed by using the comet assay. The results showed an increased number of tail length, tail DNA, olive tail moment, and tail moment in AgNPs-treated HepG2 cells. Besides, the flow cytometry analysis revealed 62.85% early apoptotic cells and 6.14% late apoptotic cells. Additionally, the cell cycle assay showed the cell cycle arrest in the sub-G_0_/G_1_ phase in AuNPs treated HepG2 cells (50). 

*Biocompatibility of* *Gold Nano-biomaterials* *: Are Gold Nano-biomaterials Safe Enough for Clinical Trials?*

Biocompatibility is an important issue for clinical therapeutic purposes to avoid systemic adverce effects (75, 76). Biocompatibility encompasses a wide range of tests and a biocompatible nanomaterial should pass these tests including genotoxicity, implantation, irritation, systemic toxicity, hemocompatibility, cytotoxicity, and sensitization (76). The biocompatibility of NPs can be affected by different parameters such as material composition, particle size, surface area, surface chemistry, and surface charge (77). The AuNPs showed no acute oral toxicity in female rats with acute oral lethal dose (LD_50_) of over 5 g/kg of body weight. Besides, the primary skin irritation test for AuNPs showed no primary irritation on rabbits (78). Furthermore, a study reported size dependent toxicity of AuNPs in mice. The mice were treated using intraperitoneal route of administration. The group treated with smaller AuNPs size distribution (3 to 5 nm) did not show any sickness, whereas fatigue, loss of appetite, and weight loss were observed for larger AuNPs (79). Moreover, in a comparative study, biologically and chemically synthesized AuNPs were compared for their toxicity on Wistar rats. The rats were orally administered with biologically and chemically synthesized AuNPs at the dose of 5 mg/kg body weight for 28 days. The results represented that the biologically synthesized AuNPs did not showed any toxicity according to the serum biochemical analysis, hematology and histochemical studies, whereas chemically synthesized AuNPs induced toxicity in Wistar rats (80). A meta analysis reported that the odds of cyototoxicity of biologically synthesized AuNPs in cancer cell lines were 6.889 times more than healthy cell lines (OR = 6.88, *P*  =  0.018) indicating far less cytotoxicity chance of biogenic AuNPs in healthy cell lines (81). However, further studies should be carried out using different tequniches to provide safety profile of biogenic AuNPs for pharmaceutical applications.

## Conclusion

This systematic review provided preliminary evidence indicating the potency of green AuNPs to combat hepatic cancer. In summary, it can be concluded that biosynthesized AuNPs had significant anticancer activity toward hepatic cancer cells through *in-vitro* models. Our findings showed that the anticancer activity of AuNPs is due to intracellular overgeneration of ROS and induction of apoptosis. Future studies should determine the anticancer potency of green AuNPs through animal studies. The studies showed that AuNPs can be used as anticancer drug nanocarrier for drug delivery systems. Moreover, AuNP surface functionalization provides an opportunity to design multifunctional nanoparticles by conjugating them to diagnostic and/or therapeutic agents for theranostic purposes. Remarkably, the synthesis of AuNPs using biosynthetic approaches does not mean that they are safe for human. An extensive toxicity assessment should be performed to determine the safety profile of AuNPs. Many challenges should be addressed in future studies including the biogenic AuNPs pharmacokinetics, pharmacodynamics, genotoxicity, immunogenicity, acute and chronic toxicity as well as the role of the protein corona on the anticancer efficacy of AuNPs and finally the fate of the AuNPs in the body.
